# Growing burden of sepsis-related mortality in northeastern Italy: a multiple causes of death analysis

**DOI:** 10.1186/s12879-016-1664-2

**Published:** 2016-07-13

**Authors:** Ugo Fedeli, Pasquale Piccinni, Elena Schievano, Mario Saugo, Giampietro Pellizzer

**Affiliations:** Epidemiological Department, Passaggio Gaudenzio 1, Padova (PD), 35131 Veneto Region Italy; Anesthesiology and Intensive Care, Eretenia Hospital, Viale Eretenio 12, 36100 Vicenza, Italy; Infectious Disease Unit, San Bortolo Hospital, Viale Rodolfi 37, 36100 Vicenza, Italy

**Keywords:** Sepsis, Mortality, Epidemiology

## Abstract

**Background:**

Few population-based data are available on mortality due to sepsis. The aim of the study was to estimate sepsis-related mortality rates and to assess the associated comorbidities.

**Methods:**

From multiple causes of death data (MCOD) of the Veneto Region (northeastern Italy), all deaths with sepsis mentioned anywhere in the death certificate were retrieved for the period 2008–2013. Among these deaths the prevalence of common chronic comorbidities was investigated, as well as the distribution of the underlying cause of death (UCOD), the single disease selected from all condition mentioned in the certificate and usually tabulated in mortality statistics. Age-standardized mortality rates were computed for sepsis selected as the UCOD, and for sepsis mentioned anywhere in the certificate.

**Results:**

Overall 16,906 sepsis-related deaths were tracked. Sepsis was mentioned in 6.3 % of all regional deaths, increasing from 4.9 in 2008 to 7.7 % in 2013. Sepsis was the UCOD in 0.6 % of total deaths in 2008, and in 1.6 % in 2013. Age-standardized mortality rates increased by 45 % for all sepsis-related deaths, and by 140 % for sepsis as the UCOD. Sepsis was often reported in the presence of chronic comorbidities, especially neoplasms, diabetes, circulatory diseases, and dementia. Respiratory tract and intra-abdominal infections were the most frequently associated sites of infection.

**Conclusions:**

MCOD analyses provide an estimate of the burden of sepsis-related mortality. MCOD data suggest an increasing importance attributed to sepsis by certifying physicians, but also a real increase in mortality rates, thus confirming trends reported in some other countries by analyses of hospital discharge records.

## Background

Few population-based data are available worldwide on the incidence and mortality from sepsis; this could be due to the fact that sepsis is a condition with many possible interactions with other disorders, and with a difficult definition within the continuum from sepsis, severe sepsis, and septic shock [1]. Available figures have mostly been obtained from analyses of hospital discharge records performed mainly in the US, in Australia, and in Northern Europe [2–4]. Different selection algorithms applied to discharge records lead to different estimates of the incidence of severe sepsis [4]. In spite of these discrepancies, all analyses confirm an increasing trend in the incidence of severe sepsis, and a decline in case fatality. Notably, the reduction in hospital mortality among patients with severe sepsis is similar to mortality time trends identified in clinical trial participants [5]. However, the rise with time in hospitalized sepsis cases is so pronounced that, in spite of the reduced case-fatality, population-based mortality in the US has been estimated to increase [6]. A recent report from Spain confirmed such tendencies, with increasing population-based mortality rates even though a decline in case-fatality was observed [7]. It must be remarked that discharge diagnoses show a limited sensitivity for detecting sepsis and severe sepsis [8]. Furthermore, doubts have been raised about a possible over-estimation of time trends from administrative data: education and care improvement campaigns, as well as reimbursement rules, could put an increasing pressure on hospital coding for sepsis [9]. Lastly, mortality figures limited to hospitalized cases could underestimate the impact of sepsis at the population level [7].

A possible alternative information source for estimating mortality from sepsis is represented by causes of mortality, which include also deaths in non-hospitalized patients. Nonetheless, conventional tabulations of mortality relying only on the underlying cause of death (UCOD) usually emphasize the burden of chronic illness, and understate the role of sepsis and infectious diseases in the terminal phase leading to death [10, 11]. A solution is represented by the analysis of all conditions mentioned in the death certificate (multiple causes of death—MCOD) [11]. Part I of the death certificate reports the causal sequence from the immediate cause of death, to intermediate causes, to a single underlying cause which initiated the train of morbid events leading directly to death; Part II of the certificate includes other significant conditions contributing to death. The UCOD is selected based on internationally adopted algorithms and generally corresponds to the underlying cause stated by the certifier, but it could also be another disease reported in Part I or II, or a derived condition. Through the MCOD approach, a more realistic estimate of the burden of mortality from sepsis can be obtained, and comorbidities reported in the certificate can be analyzed. However, to date such analyses have been carried out in few countries, with divergent findings on burden and time trends in mortality from sepsis [12–14].

The aim of the study is to analyze MCOD records in the Veneto region (northeastern Italy) to estimate sepsis-related mortality rates, to investigate infection sources leading to sepsis, and to assess the main comorbidities mentioned in sepsis-related deaths.

## Methods

The Veneto region has about 4,900,000 inhabitants; the population is rapidly ageing due to low birth rates and a life expectancy spanning to about 80 and 85 years in males and females, respectively. A copy of death certificates of each resident in the Veneto Region is routinely transmitted to the Regional Epidemiology Department for coding of the causes of death according to the International Classification of Diseases, 10th Edition (ICD-10). Since 2008 the regional mortality database includes all the diseases mentioned in the certificate; the selection of the UCOD is performed by means of the Automated Classification of Medical Entities (ACME), which is a computer program developed by the US National Center for Health Statistics to standardize assignment of the underlying cause [15].

Mention of sepsis was searched among MCOD records of the period 2008–2013 to retrieve non-surgical sepsis-related deaths, corresponding to the ICD-10 codes A02.1 (Salmonella septicaemia), A32.7 (Listerial septicaemia), A40 (Streptococcal septicaemia), A41 (Other septicaemia, including unspecified septicemia and septic shock), B37.7 (Candidal septicaemia), P36 (Bacterial sepsis of newborn). Codes A40, A41, and P36 have already been demonstrated to identify the vast majority of sepsis-related deaths with respect to broader selections [13]; the present choice of ICD-10 codes allows the comparison with the few studies investigating burden and time trends of sepsis-related mortality through analyses of MCOD data [12–14]. Based on the position where sepsis was reported in the death certificate, the following classification into mutually exclusive categories was adopted: sepsis in the line reserved for the underlying cause; sepsis not reported as the underlying cause, but mentioned in another line of Part I; and sepsis only reported in Part II of the certificate. For each position, the percentage of selection of sepsis as the UCOD by the ACME software was determined.

Proportional mortality (percentage of all registered deaths) was computed for sepsis selected as the UCOD, and for sepsis mentioned anywhere in the certificate. Proportional mortality figures were compared with those obtained through a selection of ICD-10 codes including, as well as sepsis, all deaths attributed to an infection according to the methodology adopted by Wang and colleagues [16].

Among all sepsis-related deaths, the distribution of the UCOD according to major disease categories, and the prevalence of common chronic comorbidities reported in any position of death certificates were investigated. Lastly, other ICD-10 codes were searched to identify common associated infection sources (respiratory, intra-abdominal, urinary, skin and soft tissues, Table 1).

**Table 1:** International Classification of Diseases, 10th Edition (ICD-10) codes retrieved to determine sites of infection associated to sepsis

Site	ICD-10 codes
*Respiratory tract*	
Acute upper respiratory infections	J00–J06
Influenza and pneumonia	J10–J18
Other acute lower respiratory infections	J20–J22
Chronic obstructive pulmonary disease with acute exacerbation	J440, J441
Pneumonitis due to solids and liquids	J69
Suppurative and necrotic conditions of lower respiratory tract	J85–J86
*Intra-abdominal*	
Intestinal infectious diseases	A00–A09
Appendicitis	K35–K37
Hernia	K40–K46
Vascular disorders of intestine, obstruction, diverticular disease	K55–K57
Other diseases of intestines (including perforation)	K63
Peritonitis	K65
Disorders of gallbladder and biliary tract	K80–K83
Acute pancreatitis	K85
*Skin/soft tissue*	
Erysipelas	A46
Infections of the skin and subcutaneous tissue	L00–L08
Decubitus ulcer and pressure area	L89
Ulcer of lower limb, not elsewhere classified	L97
Necrotizing fasciitis	M72.6
Gangrene, not elsewhere classified	R02
*Urinary tract*	
Acute/chronic/unspecified tubulo-interstitial nephritis	N10–N12
Cystitis	N30
Urethritis	N34
Urinary tract infection, site not specified	N390

To compute mortality rates, population data were derived from the National Institute for Statistics (http://demo.istat.it/). Age-standardized mortality rates were estimated for each year studied to explore time trends in sepsis-related mortality, with the regional population in 2008 adopted as the reference. The change in age standardized mortality rates observed in 2013 with respect to 2008 was estimated by means of the standardized Rate Ratio (RR) with the corresponding 95 % Confidence Interval (CI) [17].

Mortality data are routinely collected by the Regional Epidemiology Department; all analyses were carried out on anonymized records without any possibility of identification of individuals, therefore the study was exempt from institutional review board approval.

## Results

Sepsis was mentioned in 6.3 % of all regional deaths, increasing from 4.9 in 2008 to 7.7 % in 2013; sepsis was selected as the UCOD in only 0.6 % of all regional deaths in 2008, and in 1.6 % in 2013. The broader category of all infection-related deaths followed a similar although less pronounced trend (Table 2). The increase in proportional mortality, greater for analyses based on the UCOD compared to those relying on MCOD, was associated with a changing certification pattern: through 2008–2013, among certificates mentioning sepsis, the condition was increasingly reported in the underlying cause line, from 10 in 2008 to 19 % in 2013; a parallel rise in the percentage of selection as the UCOD was observed, from 12 to 21 % (data not shown).

**Table 2:** Sepsis and all infections selected as the underlying cause of death, and mentioned anywhere on the death certificate (multiple causes of death): number of deaths and proportional mortality (share of all registered deaths) in the Veneto Region (Italy), 2008 to 2013

	Sepsis				All infections			
	Underlying cause		Multiple causes		Underlying cause		Multiple causes	
	*n*	Proportional mortality	*n*	Proportional mortality	*n*	Proportional mortality	*n*	Proportional mortality
*Whole study period*	*2,716*	*1.0 %*	*16,906*	*6.3 %*	*14,134*	*5.2 %*	*54,919*	*20.4 %*
2008	264	0.6 %	2153	4.9 %	1,884	4.3 %	8,356	19.0 %
2009	281	0.6 %	2382	5.4 %	1,988	4.5 %	8,902	20.2 %
2010	376	0.8 %	2755	6.2 %	2,158	4.9 %	9,064	20.4 %
2011	492	1.1 %	2948	6.6 %	2,571	5.7 %	9,335	20.8 %
2012	576	1.2 %	3142	6.7 %	2,674	5.7 %	9,576	20.5 %
2013	727	1.6 %	3526	7.7 %	2,859	6.3 %	9,686	21.2 %

**Fig. 1:**
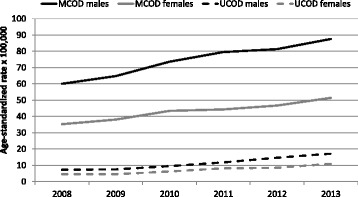
Deaths from sepsis as the underlying cause (UCOD), and mentioned anywhere on the death certificate (multiple causes, MCOD): trends in age-standardized rates per 100,000 population (standard = regional population in 2008) among male and female residents of the Veneto Region (Italy), 2008 to 2013

Figure 1 shows that mortality from sepsis increased at a similar pace in both genders; the growth in age-standardized mortality was 45 % for all sepsis-related deaths (RR = 1.45, CI 1.37–1.53), and 140 % for sepsis as the UCOD (RR = 2.40, CI 2.09–2.77). Mortality rates increased exponentially with age and remained higher in males than in females across all age classes (data not shown). The median age among sepsis-related deaths was 78 and 84 years in males and females respectively, about 1 year earlier than figures observed in overall regional deaths; median age in sepsis-related deaths increased from 80 years in 2008 to 82 years in 2013. Due to changes in variable recording through the study period, place of death was available only in 2013 for about 97 % of regional deaths; although the vast majority of deaths with sepsis mentioned as MCOD happened in acute care hospitals (Table 3), a non-negligible proportion affected patients in hospice and long-term care facilities.

**Table 3:** Characteristics of 16,906 deaths with mention of sepsis anywhere on the death certificate: Veneto Region (Italy), 2008–2013

	% of all sepsis-related deaths
Male sex	50.0
Age	
<65 yrs	12.6
65–74 yrs	16.9
75–84 yrs	34.5
≥85 yrs	35.9
Died in Hospital^a^	91.1
Underlying cause of death (ICD-10 codes)	
Sepsis (A02.1,A32.7,A40, A41,B37.7,P36)	16.1
Circulatory diseases (I00–I99)	17.4
Neoplasms (C00–D48.9)	20.9
Digestive diseases (K00–K99)	11.7
Respiratory diseases (J00–J99)	11.1
Dementia, Alzheimer (F01–F03, G30)	6.0
Other diseases	16.8
Comorbidities (ICD-10 codes)	
Neoplasms (C00–D48.9)	26.9
Diabetes (E10–E14)	14.6
Ischemic heart diseases (I20–I25)	14.5
Hypertensive diseases (I10–I15)	12.7
Cerebrovascular diseases (I60–I69)	14.7
Dementia, Alzheimer (F01–F03, G30)	11.6
COPD (J40–J44, J47)	6.3
Chronic liver diseases (B15–B19,C22,K70,K73,K74)	5.2
Chronic renal failure (N18.5, N18.9)	7.3
HIV (B20–B24, R75)	0.3
Associated sites of infection	
Respiratory tract	27.5
Gastrointestinal tract	16.6
Skin/soft tissue	8.6
Urinary tract	5.7

^a^3424 deaths in 2013 with available data

In most sepsis-related deaths another underlying cause was selected, distributed across the main nosological sectors. According to MCOD analyses, sepsis was often mentioned in the presence of chronic comorbidities such as neoplasms, diabetes, specific circulatory diseases, and dementia (Table 3).

Lastly, Table 3 reports the main sites of infection retrieved in sepsis-related deaths: respiratory tract and intra-abdominal infections were the most cited. Taking into account that in the same certificate more than one site could be mentioned, in about 47 % of deaths none was identifiable. Among these latter deaths, most had sepsis (28 %) or cancer (29 %) as the UCOD.

## Discussion

Sepsis is selected as the UCOD only in a limited fraction of death certificates where the condition is mentioned; MCOD analyses capture a greater burden of sepsis-related mortality. In the present study, in 2013 sepsis was the UCOD in 1.6 % of overall deaths whereas the condition was mentioned in 8 % of death certificates. Moreover, by means of MCOD a more realistic estimate of time trends could be obtained. Mortality figures based on the UCOD depend both on mention of sepsis among multiple diseases cited in the death certificate, and on the position where sepsis is reported in the certificate, with the associated probability of selection as the UCOD (highest on the underlying cause line). Among death certificates with mention of sepsis, the proportion with sepsis on the underlying cause line is growing over time, possibly due to the increasing awareness about early recognition and treatment of sepsis, and to the increasing importance attributed to the role of sepsis in the pathway leading to death. Due to these changing certification practices, across the study period mortality rates from sepsis based on the UCOD increased much more than those based on MCOD.

Sepsis-related mortality rates based on MCOD were reported to be roughly stable from 1999 to 2005 in the US [12], and from 2001 to 2010 in England [13], while a steep increase has been registered in Brazil from 2002 to 2010 [14]. Similarly to discharge records, it remains uncertain if observed time trends are a real epidemiological phenomenon or rather an artifact due to changes in coding practices of administrative archives. However, it must be remarked that mortality records are not collected for reimbursement, but only for epidemiological and public health purposes. Although we lack validation of death certificates reporting sepsis, a reasonable interpretation of the present findings is that the huge increase in rates based on the UCOD is an overestimation due to time changes in the importance attributed to the condition, whereas the more limited but still large increase in rates based on MCOD probably reflects a real epidemiological trend, as suggested also by the increase in proportional mortality figures for all infections combined.

Ageing of the regional population with the associated increase in multiple chronic comorbidities is probably the main force leading to the growth of sepsis-related mortality. Although age among sepsis-related deaths is slightly lower than age at death in the overall regional population, it is worth noting that about 35 % of decedents with sepsis mentioned in the certificate were aged ≥85 years; furthermore, median age at death increased through the study period. It must be remarked that among sepsis-related deaths a higher prevalence of comorbidities such as cancer or diabetes could be observed in the present study with respect to MCOD analyses carried out in other countries [12–14]; this observation could be explained by the study population being shifted toward older age classes. According to analyses of US discharge records, among patients hospitalized for infections there was an increasing trend in the prevalence of comorbidities through 2003–2009, reflecting a changing population more susceptible to sepsis [18]. This trend has been confirmed by analyses of hospitalizations in Spain, demonstrating that over time cases of severe sepsis occurred in older people who had more comorbidities, painting a picture of greater frailty and severity of disease over time [7]. Nonetheless, the demographic and epidemiological context does not make the increase in sepsis-related mortality an unchangeable trend, since opportunities to improve cardiovascular and oncological mortality in the elderly may also lay in better treatment of infection and severe sepsis [10, 19]. In fact, in a population-based study on hospital data from Taiwan, a nationwide education program on clinical practice in sepsis was associated with a decrease of in-hospital mortality [20].

Analysis of sepsis-related mortality by means of mortality records has a number of limitations. First, the choice of ICD-10 codes varies between studies, but the selected codes were demonstrated to identify the majority of sepsis-related deaths [13]. Furthermore, reporting sepsis in Part II of the death certificate (outside the causal chain directly leading to death) can be considered inappropriate; in the present study the percentage of such death certificates was limited to about 5 %, and stable over time. Lastly, the associated site infection is often missing, especially in patients with cancer or when the certifiers choose to mention directly sepsis on the underlying cause line. In a multicenter study of patients with septic shock in intensive care units, the most common anatomic source of infection was the lung (40 %), followed by intra-abdominal (31 %) and genitourinary tract infections (11 %); there was great variation in mortality by source [21]. Urinary tract infections showed the lowest in-hospital mortality; the intra-abdominal site included infections with both low (enterecolitis, cholecystitis) and high mortality (ischemic bowel), that were all grouped together in the present study. In spite of the incomplete and row classification, MCOD could help to monitor time trends in infection sources of sepsis.

## Conclusions

MCOD can be considered a useful tool to estimate the burden of sepsis-related mortality, to confirm time trends depicted by other information sources such as discharge records, to investigate the associated sites of infection, and to assess the role of chronic comorbidities.

MCOD analyses suggest that, beyond an increasing importance attributed to sepsis by certifying physicians, an increase in sepsis-related mortality rates can be observed, possibly due to ageing of the population with the associated burden of multiple chronic diseases.

## Abbreviations

ACME, Automated Classification of Medical Entities; ICD-10, International Classification of Diseases, 10th Edition; MCOD, multiple causes of death; UCOD, underlying cause of death
